# Clinical, laboratory, and medication-related factors associated with in-hospital mortality among oncology patients at a tertiary hospital in Saudi Arabia

**DOI:** 10.3389/fphar.2026.1825631

**Published:** 2026-08-28

**Authors:** Ahmed Alafnan, Meshal M. Alorainan, Sanad M. Alanazi, Salman F. Algharbi, Faisal R. Alshammari, Ammar Alghaslan, Hammad Saleem, Saleh Alghamdi, Mohd Farooq Shaikh, Sirajudheen Anwar

**Affiliations:** 1 Department of Pharmacology and Toxicology, College of Pharmacy, University of Hail, Hail, Saudi Arabia; 2 College of Pharmacy, University of Hail, Hail, Saudi Arabia; 3 Institute of Pharmaceutical Sciences (IPS), University of Veterinary and Animal Sciences (UVAS), Lahore, Pakistan; 4 Department of Clinical Pharmacy, Faculty of Pharmacy, Al-Baha University, Al-Baha, Saudi Arabia; 5 School of Dentistry and Medical Sciences, Charles Sturt University, Orange, NSW, Australia

**Keywords:** hospitalized cancer patients, hypo-albuminemia, in-hospital mortality, laboratory biomarkers, medication safety, oncology, polypharmacy, potential drug–drug interactions

## Abstract

**Background:**

Oncology inpatients face physiological decline alongside complex pharmacology, yet clinical mortality predictors and drug-related risks remain understudied in Middle Eastern populations. This study identified independent predictors of in-hospital mortality and characterized potential drug–drug interactions (pDDIs) among hospitalized cancer patients in Saudi Arabia.

**Methods:**

This retrospective cohort study evaluated 401 adult oncology inpatients admitted to a tertiary care center in Hail, Saudi Arabia (2020–2022). Demographics, clinical parameters, and medication profiles were extracted from medical records. Bivariate correlations were assessed using point-biserial ( $r_{pb}$ ) and phi ( ϕ ) coefficients. Independent mortality predictors were identified via multivariable binary logistic regression, and biomarker discrimination was evaluated using receiver operating characteristic (ROC) curves. Potential DDIs were screened using Drugs.com.

**Results:**

The cohort was predominantly female (67.6%) and Saudi (85.0%), with solid malignancies accounting for 94.3% of cases. In-hospital mortality was 25.2% ( n=101/401 ). Bivariate correlations demonstrated significant associations with mortality across clinical parameters, including mechanical ventilation requirements ( ϕ=0.118,p=0.018 ). Multivariable regression identified four independent physiological predictors of mortality: renal failure ( aOR=44.29,95% CI:1.07−1836.31 ), baseline albumin $\left.\text{aOR}=0.71\text{ per }g/L,95\%\text{ CI}:0.55-0.91\right)$ , serum creatinine ( aOR=1.02 per μmol/L,95% CI:1.001−1.04 ), and oxygen saturation ( aOR=0.68 per %,95% CI:0.50−0.93 ). Serum albumin showed high individual discrimination ( AUC=0.920 ), while the combined multivariable model achieved an AUC of 0.953. Pharmacological screening revealed near-universal interaction prevalence: 98.6% of 2,148 prescriptions contained a pDDI flag (54.9% severe), and 96.5% of patients had ≥1 severe pDDI flag.

**Conclusion:**

In-hospital mortality is strongly driven by acute organ dysfunction and physiological decline rather than database-flagged interactions alone. However, the high prevalence of severe pDDI flags highlights a critical need to integrate structured clinical pharmacy reviews into oncology admission protocols. Targeted medication safety interventions should focus on avoiding nephrotoxic combinations and establishing pre-therapeutic pharmacogenetic screening to mitigate potential iatrogenic risks.

## 1 Introduction

Cancer remains a leading cause of premature death worldwide, posing one of the greatest continuing public health challenges of our time. According to one survey carried out by the World Health Organization (WHO), people in most countries can expect cancer to be ranked among the top three causes of death before reaching 70 years of age (WHO, 2020). In 2020, nearly 10 million people died from cancer globally, and this figure is projected to exceed 16 million per year by 2040, driven primarily by population ageing, tobacco exposure, obesity, and metabolic disturbances (Sung et al., 2021; Arnold et al., 2022). In many developing and middle-income nations, tobacco consumption, rising obesity rates, and metabolic comorbidities have substantially altered cancer epidemiological patterns over recent decades (Islami et al., 2024).

Cancer patients constitute one of the most pharmacologically complex populations in contemporary clinical practice. Beyond cytotoxic chemotherapy, they typically receive corticosteroids, antiemetics, proton pump inhibitors, anticoagulants, antibiotics, antifungals, analgesics, and various supportive care agents concurrently. This polypharmaceutical environment creates a substantial risk of clinically meaningful drug - drug interactions (DDIs). Most antineoplastic agents - taxanes and platinum-based regimens in particular - are metabolised through cytochrome P450 pathways, rendering them vulnerable to pharmacokinetic interactions with co-prescribed drugs (Cheng et al., 2022). Additive toxicity through pharmacodynamic mechanisms, including nephrotoxicity, myelosuppression, and QT prolongation, compounds this risk further and is difficult to anticipate without systematic medication review. Hospitalised cancer patients carry a higher burden of adverse drug events than non-oncology populations, a pattern attributable in part to polypharmacy and cumulative DDI exposure (Cheng et al., 2022).

Despite progress in prevention and treatment, clinical outcomes at discharge vary considerably across populations, attributed to differences in age distribution, comorbidity burden, and healthcare access (Chien et al., 2023). Available evidence suggests that age, organ dysfunction, and the presence of significant comorbidities are among the key determinants of cancer prognosis (Wang et al., 2025). Specifically, conditions such as diabetes mellitus, renal failure, and hypertension accelerate disease progression and frequently limit the delivery of full therapeutic doses, thereby impacting overall discharge status (Chaudhri et al., 2020; Rajabu et al., 2024). Additionally, smoking remains a well-established independent predictor of in-hospital mortality across multiple cancer types (Del Riccio et al., 2022). Over the past 2 decades, both incidence and the mortality proportion from cancer have risen steadily in Saudi Arabia (Alsuhaymi et al., 2024). Colorectal, breast, and hematologic malignancies account for the greatest clinical burden, yet vital status at discharge within each category depends considerably on the individual patient profile and associated risk factors (Almatroudi, 2020; Shurrab et al., 2022). Prior work has concentrated largely on site-specific cancers, leaving a gap in the evidence base for studies that assess these short-term clinical outcomes under the combined influence of demographic and clinical variables (Albasri and Ansari, 2021).

While numerous studies have evaluated mortality predictors in critically ill patients, fewer have focused specifically on hospitalised oncology patients outside intensive care settings. Mortality in this population is multifactorial: infection, organ dysfunction, treatment-related toxicity, and disease progression each contribute in varying degrees. Identifying modifiable predictors, such as laboratory derangements or medication-related risk factors, may offer opportunities for earlier intervention (Costantini and Budillon, 2020). Evaluating both clinical and pharmacological determinants of in-hospital mortality therefore provides a more complete basis for risk stratification in this patient group.

Although previous research has increasingly recognized the clinical burden of potential drug-drug interactions (pDDIs) in oncology, literature characterizing these events remains skewed toward specific care environments. Historically, investigations into inpatient drug safety have primarily focused on critical care or intensive care settings, where complex medication changes are standard (Obeid and Karara, 2022). In contrast, studies targeting non-intensive care unit (non-ICU) hospitalized oncology patients are relatively sparse, spanning isolated international and regional cohorts. Prior regional assessments have evaluated the prevalence and distinct predictors of pDDIs in general inpatient oncology wards, identifying advanced age, prolonged length of hospital stay, the presence of hematologic malignancies, and underlying polypharmacy as prominent drivers of exposure (Tavakoli-Ardakani et al., 2013; Wondm et al., 2023). Furthermore, observed clinical outcomes in these populations range from prolonged hospitalization to severe toxicities, such as dangerous QT-interval prolongations resulting from antiemetic and systemic chemotherapy co-prescription (Turossi-Amorim et al., 2022).

However, these existing studies suffer from collective limitations; they are frequently restricted by single-center designs, small sample sizes, or a narrow focus on exclusive drug classes—such as interactions driven solely by antineoplastic regimens or supportive care agents. Crucially, there is a distinct lack of comprehensive literature integrating full profile pDDI screening data that captures the overlapping use of chemotherapy, supportive medications, and drugs managed for concurrent, non-oncological comorbidities within general oncology floors. This omission creates a significant knowledge gap, as it leaves the full scope of real-world medication risk unquantified for hospitalized cancer patients outside the ICU.

To address this gap, the present study retrospectively analyzed 401 adult oncology inpatients treated at a tertiary referral center in Hail, Saudi Arabia. This study is an exploratory observational analysis designed to identify clinical, laboratory, and medication-related factors (specifically the prevalence and severity of potential DDIs) associated with in-hospital mortality among non-ICU hospitalized oncology patients, and to evaluate whether these pDDIs are independently associated with mortality.

## 2 Materials and methods

### 2.1 Study design and setting

This retrospective analytical cohort study was conducted at King Salman Hospital, a tertiary care academic medical center and regional referral hub in the Hail region, Saudi Arabia. The oncology service at this facility features a dedicated to inpatient ward. Electronic health records were systematically reviewed over a 3-year calendar period, from 1 January 2022, to 31 December 2024, to identify baseline clinical, laboratory, and pharmacological predictors (including potential drug-drug interactions [pDDIs]) of in-hospital mortality among adult cancer patients. Eligible patients were anchored at hospital admission (“Time Zero”), and baseline predictors were defined as the first clinical or laboratory measurements obtained within 24 h of admission. Post-admission longitudinal data, such as subsequent laboratory fluctuations or mechanical ventilation events, were isolated from the baseline framework and analyzed as markers of hospital-course severity through to hospital discharge. The institutional ethics committee reviewed and approved the study protocol, which was conducted in strict accordance with the Declaration of Helsinki and national guidelines for biomedical research (Alotaibi et al., 2022).

### 2.2 Participant selection and eligibility

**FIGURE 1:**
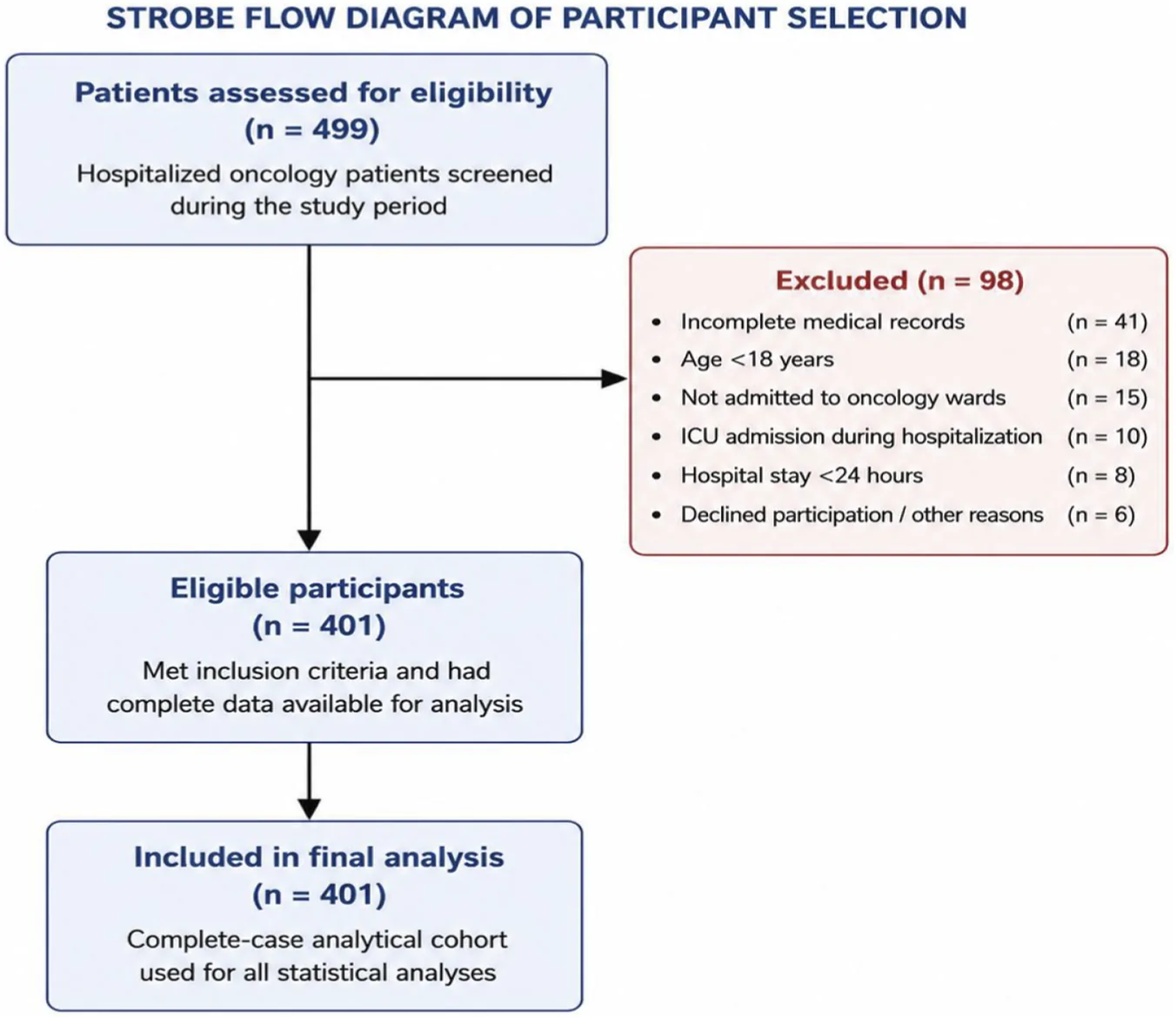
STOBE flow diagram of participant selection. A total of 499 hospitalized oncology patients were screened for eligibility. Following the application of predefined exclusion criteria, 98 patients were excluded. The final analytical cohort comprised 401 patients who were included in all statistical analyses.

A total of 499 hospitalized oncology patients were initially screened for study eligibility. Of these, 98 patients were excluded for the following reasons: incomplete potential drug-drug interaction (pDDI) screening data ( n=41 ), age under 18 years ( n=18 ), a short hospital stay of less than 48 h ( n=15 ), ICU admission during the study period ( n=10 ), absence of systemic antineoplastic therapy ( n=8 ), or declined participation and other reasons ( n=6 ). Consequently, 401 patients met all eligibility criteria and were enrolled, establishing a final analytical cohort of 401 patients with integrated, comprehensive pDDI data (Figure 1). The cohort included both solid and hematological malignancies and comprised Saudi and non-Saudi nationals, reflecting the mixed patient population of the study center.

### 2.3 Operational definitions

To ensure reproducibility, the following criteria were applied during cohort selection:

Proven Malignancy: Histopathologically or cytologically confirmed solid tumors or hematological malignancies. Supportive/Palliative Care Admission: Non-curative hospitalizations intended strictly for symptom management, pain control, or end-of-life care, rather than active tumor-directed therapeutic interventions. Complete Data: Having full electronic health record documentation for baseline vital signs, initial laboratory panels, and active medication reconciliation sheets at the index admission.

### 2.4 Data collection and variables

Data were extracted from the hospital electronic health record system and the institutional cancer registry using a standardized data-extraction form. Demographic variables collected included age, sex, and nationality. Comorbid conditions recorded at baseline were diabetes mellitus, hypertension, renal disease (defined as a documented diagnosis of nephropathy or a baseline serum creatinine above the institutional upper reference limit), pulmonary disease, cardiac disease, and hypothyroidism. Smoking status was recorded as a lifestyle variable. Clinical variables included cancer type, disease extent, anticancer therapy profiles, and length of hospital stay. To prevent outcome leakage, all baseline laboratory parameters—including aspartate aminotransferase (AST), alanine aminotransferase (ALT), serum albumin (hypoalbuminemia defined as <35 g/L), total protein, serum creatinine, hemoglobin, red blood cell count, white blood cell count, international normalized ratio (INR), blood pressure measurements, and oxygen saturation—were strictly restricted to the first measurement obtained within 24 h of hospital admission (“Time Zero”). Polypharmacy was defined as five or more medications prescribed concurrently during the index hospitalization. Post-admission events, such as requirements for mechanical ventilation and in-hospital discharge status, were tracked through to discharge and isolated as markers of hospital-course severity (Basudan et al., 2023).

### 2.5 Data validation and quality control

Data were extracted using a structured abstraction form applied systematically across all medical records. Baseline laboratory values were taken from results obtained on admission; where multiple values were available within the first 24 h, the first documented result was used. Medication lists were cross-checked against physician prescribing orders and nursing administration charts to minimize transcription error. To ensure data integrity, a PharmD investigator conducted the data extraction, and a random 10% sample ( n=40 ) of all charts was independently double reviewed by the Principal Investigators. Inter-rater reliability was formally assessed using Cohen’s kappa, yielding an excellent agreement score ( κ=0.88 ). Remaining discrepancies were documented and resolved through consensus review involving a third senior investigator

### 2.6 Drug interaction analysis

Potential drug-drug interactions (pDDIs) were systematically evaluated using the web-based Drugs.com Drug Interaction Checker (Drugsite Trust, Auckland, New Zealand; accessed 2024–2026). This platform functions as a dynamic interface that integrates clinical interaction data from three independent reference engines: IBM Micromedex (Truven Health Analytics, Greenwood Village, CO, United States), the Cerner Multum Drug Information Suite (Cerner Corporation, Kansas City, MO, United States), and the American Society of Health-System Pharmacists (ASHP) Drug Interactions Database (ASHP, Bethesda, MD, United States). The utility of this tool as a screening instrument in retrospective oncology research is supported by a published evaluation of nine DDI screening tools across 145 oral oncology drug pairs, in which Drugs.com achieved a performance score of 0.83, with no statistically significant difference compared to the top-performing subscription-based tool, Lexicomp® (0.89) (Marcath et al., 2018). For each drug prescribed during the index admission, the following screening outputs were recorded in a structured database: The potential interaction type, the name of the interacting drug partner (where identified), the potential clinical effect, the screening severity score, the evidence level. Screening severity was classified on a four-point scale: 0 = no significant interaction, 1 = moderate, 2 = severe, and 3 = most severe. Potential interaction types were categorized under one of the following mechanisms or risks: metabolic interaction, toxicity overlap, nephrotoxicity, cardiotoxicity, neuropathy, hepatotoxicity, immune-related toxicity, thrombosis risk, or central nervous system (CNS) depression. Organ-specific interaction flags were recorded at the drug prescription level for potential nephrotoxicity, hepatotoxicity, neuropathy, cardiotoxicity, and thrombosis risk. These were then aggregated to the patient level to determine whether a patient was prescribed at least one medication carrying that specific potential interaction class. Finally, the prevalence of each organ-specific potential interaction category was compared between survivors and non-survivors using the chi-square test, or Fisher’s exact test where expected cell counts fell below five, to evaluate statistical associations with mortality.

### 2.7 Handling of confounding variables

Potential confounding factors were taken into account, which were dependent on clinical plausibility and previous literature. Variables entering the multivariable model were selected on the basis of statistical significance in univariate analysis (p < 0.05) or known biological plausibility from existing literature. Multicollinearity was assessed using Pearson correlation matrices and variance inflation factors. Highly correlated variables were not entered simultaneously into the regression model.

### 2.8 Receiver operating characteristics analysis

The discriminatory ability of individual laboratory biomarkers and the combined multivariable logistic regression model to predict in-hospital mortality was assessed using receiver operating characteristic (ROC) curve analysis. The area under the ROC curve (AUC) was calculated for serum albumin, serum creatinine, INR, and oxygen saturation as individual predictors. For continuous variables where higher values indicate lower risk, the variable sign was reversed before ROC analysis to ensure the AUC reflected predictive performance in the direction of mortality. The optimal cut-off value for each biomarker was determined using the Youden index (J = sensitivity + specificity − 1). The combined-model ROC curve was derived from the predicted probabilities of the multivariable logistic regression model, fitted in complete-case analysis to all patients with available data on the five predictor variables (n = 305 of 401 patients). AUC values of 0.70–0.80 represent acceptable discrimination, 0.80–0.90 excellent discrimination, and above 0.90 outstanding discrimination. ROC analysis was performed using Python (scikit-learn version 1.3). All AUC values are reported alongside the Youden-optimal cut-off, sensitivity, and specificity.

### 2.9 Statistical analysis

Continuous variables were assessed for normality of distribution using the Shapiro–Wilk test alongside visual inspection of normal Q–Q plots. Normally distributed continuous data are presented as mean ± standard deviation (SD) and compared between groups using the independent-samples t -test. Non-normally distributed continuous variables are expressed as median [interquartile range: Q1, Q3] and evaluated using the non-parametric Mann–Whitney U test. Categorical variables are expressed as frequencies and percentages ( n,% ) and compared using the Chi-square ( $\chi^{2}$ ) test, or Fisher’s exact test if any expected contingency cell count fell below 5.

Continuous and categorical clinical parameters were systematically evaluated for completeness prior to statistical modeling. Missing data were observed in select clinical parameters, most notably oxygen saturation ( 22.4% missingness) and laboratory indices, concentrated among patients admitted in acute distress. To prevent sample bias and maintain model stability, multivariable binary logistic regression and ROC curve analyses were conducted using a complete-case analysis approach, resulting in an analytical sample of n=305 patients with complete records across all model predictors. Variable-level missingness counts and percentages are detailed in Supplementary Table S1

Bivariate associations with the binary outcome of in-hospital mortality ( 0=Survivor ; 1=Deceased ) were evaluated using point-biserial correlation ( $r_{pb}$ ) for continuous variables and the phi coefficient ( ϕ ) for dichotomous categorical parameters.

To identify independent predictors of in-hospital mortality, a multivariable binary logistic regression model was constructed. Candidate variables were selected *a priori* based on a clinical conceptual framework and known pathophysiological risks (including organ-specific potential drug–drug interaction (pDDI) exposures) rather than relying solely on automated univariable screening thresholds to ensure model stability. The underlying assumptions of the logistic regression model were rigorously evaluated: logit linearity for continuous independent variables was verified using Box–Tidwell transformations, and multicollinearity was ruled out via Variance Inflation Factors ( VIF<2.5 ). Extreme or influential observations were screened using Cook’s distance, confirming that no individual case exceeded the operational threshold of 1.0.

Multivariable regression results are expressed as adjusted odds ratios ( aORs ) with corresponding 95% confidence intervals ( CIs ). Overall model calibration and fit were assessed using the Nagelkerke pseudo- $R^{2}$ and the Hosmer–Lemeshow goodness-of-fit test ( p>0.05 ). The discriminatory performance of individual clinical predictors and the final combined model was evaluated using receiver operating characteristic (ROC) curve analysis to determine the area under the curve ( AUC /C-statistic), with optimal cut-off values determined by the Youden index ( J=Sensitivity+Specificity−1 ). All statistical analyses were conducted using IBM SPSS Statistics (Version 27.0; IBM Corp., Armonk, NY, United States). A two-sided p<0.05 was considered statistically significant across all evaluations (Sung et al., 2021).

## 3 Results

### 3.1 Baseline demographic and clinical characteristics

The cohort comprised 401 patients, of whom 300 (74.8%) were discharged alive and 101 (25.2%) experienced in-hospital mortality. The population was predominantly female (67.6%) and Saudi (85.0%), though male patients exhibited a notably higher mortality rate than females (36.2% vs. 19.9%). Solid malignancies accounted for the vast majority of cases (94.3%), and patients with metastatic (Stage IV) disease faced a starkly elevated mortality rate of 51.3%, compared to 18.9% for those with non-metastatic cancer. Chronic comorbidities were highly prevalent, led by hypertension (40.4%) and diabetes mellitus (33.7%); however, renal disease (13.5% prevalence) and pulmonary disease (20.2% prevalence) presented the highest subgroup mortality risks at 48.1% and 34.6%, respectively. Additionally, while current or ex-smokers constituted only 5.0% of the cohort, they experienced a substantial mortality rate of 45.0% (Table 1).

**TABLE 1:** Baseline demographic and clinical characteristics of the patient cohort stratified by discharge and mortality status ( n=401 ).

Characteristic	Full cohort (N = 401) n (%)	Discharged alive (n = 300) n (%)	In-hospital mortality (n = 101) n (%)
Sex			
Female	271 (67.6)	217 (80.1)	54 (19.9)
Male	130 (32.4)	83 (63.8)	47 (36.2)
Nationality			
Saudi	341 (85.0)	256 (75.1)	85 (24.9)
Non-Saudi	60 (15.0)	44 (73.3)	16 (26.7)
Cancer type			
Solid malignancy	378 (94.3)	279 (73.8)	99 (26.2)
Hematologic malignancy	23 (5.7)	21 (91.3)	2 (8.7)
Disease extent			
Non-metastatic	323 (80.5)	262 (81.1)	61 (18.9)
Metastatic (Stage IV)	78 (19.5)	38 (48.7)	40 (51.3)
Comorbidities			
Hypertension	162 (40.4)	119 (73.5)	43 (26.5)
Diabetes mellitus	135 (33.7)	99 (73.3)	36 (26.7)
Pulmonary disease	81 (20.2)	53 (65.4)	28 (34.6)
Cardiac disease	76 (19.0)	55 (72.4)	21 (27.6)
Renal disease	54 (13.5)	28 (51.9)	26 (48.1)
Smoking status			
Non-smoker	381 (95.0)	289 (75.9)	92 (24.1)
Current/ex-smoker	20 (5.0)	11 (55.0)	9 (45.0)

Data are expressed as number (percentage). Total cohort percentages are calculated based on the full sample ( n=401 ). Survival status percentages (Discharged Alive vs. In-Hospital Mortality) are calculated horizontally based on the row total for each specific baseline characteristic.

### 3.2 Bivariable associations with in-hospital mortality

Bivariable analyses evaluating clinical and demographic factors against vital status at discharge are summarized in Table 2. Bivariate analysis revealed that disease extent, renal disease, biological sex, and pulmonary disease were statistically significant predictors of in-hospital mortality ( p<0.05 ). The strongest clinical correlations were observed for metastatic disease and baseline renal disease (both p<0.001 ), which demonstrated starkly elevated subgroup mortality rates of 51.3% (vs. 18.9% for non-metastatic) and 48.1% (vs. 21.6% for absent), respectively. Male sex was also highly associated with increased mortality compared to female sex (36.2% vs. 19.9%; p=0.001 ), while baseline pulmonary disease carried a significantly higher mortality risk of 34.6% (vs. 22.8%; p=0.042 ). Conversely, smoking status showed an adverse clinical trend that did not reach statistical significance, likely due to small subgroup size (45.0% vs. 24.1%; p=0.067 ), and cancer type demonstrated no significant correlation with survival outcomes ( p=0.103 ).

**TABLE 2:** Association between selected demographic and clinical variables and in-hospital mortality ( N=401 ).

Variable	Category	Total (N)	Mortality n (%)	Test applied	Exact p-value
Sex	Female Male	271 130	54 (19.9) 47 (36.2)	Pearson $\chi^{2}$	0.001
Cancer type	Solid Hematologic	378 23	99 (26.2) 2 (8.7)	Fisher’s exact	0.103
Disease extent	Metastatic Non-metastatic	78 323	40 (51.3) 61 (18.9)	Pearson $\chi^{2}$	<0.001
Renal disease	Present Absent	54 347	26 (48.1) 75 (21.6)	Pearson $\chi^{2}$	<0.001
Pulmonary disease	Present Absent	81 320	28 (34.6) 73 (22.8)	Pearson $\chi^{2}$	0.042
Smoking status	Current/ex-smoker Non-smoker	20 381	9 (45.0) 92 (24.1)	Fisher’s exact	0.067

Percentages represent row-wise in-hospital mortality within each variable category. Statistical significance was evaluated at the α=0.05 level. *Test Applied*: Pearson indicates Pearson’s Chi-squared test; Fisher’s Exact indicates Fisher’s exact test used for subgroups with small, expected cell counts ( n<5 ).

### 3.3 Continuous clinical parameters and survival outcomes

Table 3 evaluates normally distributed continuous clinical parameters between patients who were discharged alive ( n=300 ) and those who experienced in-hospital mortality ( n=101 ). Patients who died in the hospital were significantly older (mean 62.2±15.7 vs. 57.5±14.8 years; p=0.007 ) and presented with profoundly lower mean hemoglobin ( 9.46±1.87 vs. 11.84±2.02 g/dL; p<0.001 ) and red blood cell counts ( 3.31±0.72 vs. $4.16\pm 0.73\times 10^{6}$ /µL; p<0.001 ). Hemodynamic markers were also severely compromised in the mortality group, characterized by substantially reduced left systolic blood pressure ( 89.4±32.4 vs. 124.4±20.6 mmHg; p<0.001 ), right systolic blood pressure ( 108.8±25.1 vs. 124.2±24.3 mmHg; p<0.001 ), and right diastolic blood pressure ( 63.8±15.8 vs. 71.8±11.1 mmHg; p<0.001 ). Methodological rigor is underscored by the inclusion of 95% confidence intervals and Cohen’s d effect sizes, which reveal that left systolic BP, hemoglobin levels, and red blood cell counts exhibited the most pronounced magnitude of clinical effect between the two groups (Cohen’s d>1.10 ).

**TABLE 3:** Comparison of baseline hemodynamic, hematological, and demographic parameters by patient discharge status.

Variable	Alive (mean ± SD) (n = 300)	Dead (mean ± SD) (n = 101)	Mean diff. (95% CI)	Cohen’s d	p-value
Age (years)	57.5 ± 14.8	62.2 ± 15.7	−4.70 (−8.21 to −1.19)	0.31 (small)	0.007
Haemoglobin (g/dL)	11.84 ± 2.02	9.46 ± 1.87	2.38 (1.93–2.83)	1.20 (Very large)	<0.001
Red blood cells ( $\times 10^{6}$ /µL)	4.16 ± 0.73	3.31 ± 0.72	0.85 (0.69–1.01)	1.17 (Very large)	<0.001
Left systolic BP (mmHg)	124.4 ± 20.6	89.4 ± 32.4	35.00 (29.21–40.79)	1.45 (huge)	<0.001
Right systolic BP (mmHg)	124.2 ± 24.3	108.8 ± 25.1	15.40 (9.92–20.88)	0.63 (medium)	<0.001
Right diastolic BP (mmHg)	71.8 ± 11.1	63.8 ± 15.8	8.00 (5.08–10.92)	0.64 (medium)	<0.001

Data are presented as Mean ± SD. Group sizes are n=300 for the “Alive” cohort and n=101 for the “Dead” cohort. Mean differences (Alive minus Dead) are presented with their corresponding 95% confidence intervals (CI). Cohen’s d was calculated using pooled standard deviations to estimate effect size magnitude ( 0.2=small ; 0.5=medium ; 0.8=large ; >1.2=very large/huge ). Two-sample independent t -tests were applied for all between-group comparisons.

### 3.4 Comparison of non-normally distributed clinical and laboratory parameters by discharge status

**FIGURE 2:**
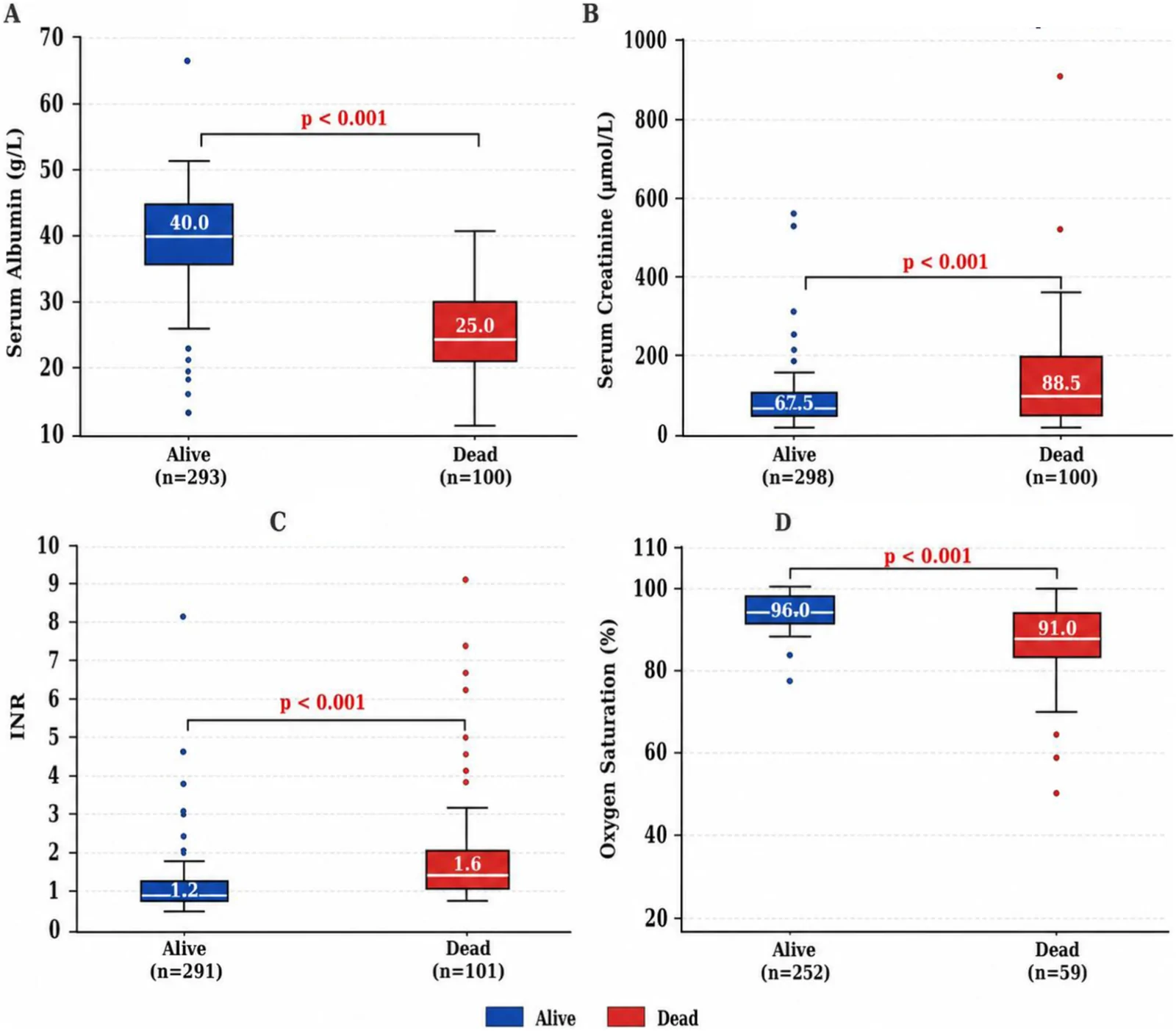
Key laboratory biomarkers by discharge outcome.Comparison of clinical parameters between survivors (Alive, blue) and non-survivors (Dead, red): **(A)** Serum Albumin (g/L), **(B)** Serum Creatinine (µmol/L), **(C)** International Normalized Ratio (INR), and **(D)** Oxygen Saturation (%). Boxes indicate the interquartile range (IQR, 25th–75th percentiles) with median values marked by white horizontal lines and numeric labels. Whiskers extend to 1.5 × IQR from the lower (Q1) and upper (Q3) quartiles; individual points represent outliers. Statistical significance was determined using the two-sided Mann–Whitney U test (p < 0.001 across all panels). n denotes the number of available observations per subgroup.

Non-survivors experienced significantly longer hospital stays and were characterized by elevated baseline levels of aspartate aminotransferase (AST), alanine aminotransferase (ALT), creatinine, white blood cell (WBC) count, and international normalized ratio (INR). Conversely, patients who died exhibited significantly lower levels of albumin, total protein, oxygen saturation ( $SpO_{2}$ ), and diastolic blood pressure. No significant differences in body temperature or respiratory rate were observed between the groups (Table 4; Figure 2).

**TABLE 4:** Comparison of non-normally distributed clinical and laboratory parameters by discharge status.

Variable	Alive median [Q1–Q3]	Dead median [Q1–Q3]	p value
Hospital stay (days)	1 [0–4]	5 [1, 1–4]	<0.001
AST (U/L)	21 [15–30]	54 [24–205]	<0.001
ALT (U/L)	19 [13–31]	28 [13–74]	<0.001
Total protein (g/L)^a^	68 [63–72]	62 [53–69]	0.001
Albumin (g/L)	40 [36–43]	25 [22–30]	<0.001
Creatinine (µmol/L)	64.5 [53–79]	88.5 [49–170]	<0.001
WBC (×10^9^/L)	6.4 [4.8–8.5]	10.5 [6.3–16.1]	<0.001
INR	1.1 [1.1–1.2]	1.6 [1.3–2.0]	<0.001
Oxygen saturation (%)^b^	97 [95–98]	91 [85–95]	<0.001

Data are presented as median [Q1–Q3] to reflect the interquartile range (25th and 75th percentiles). Base sample sizes are n=300 for the “Alive” cohort and n=101 for the “Dead” cohort, unless otherwise indicated. Between-group comparisons were evaluated using the non-parametric Mann–Whitney U test.

^a^ Total protein values were available for a subset of 146 alive and 65 dead patients.

^b^ Oxygen saturation values were available for 252 alive and 59 dead patients (reflecting a 22.4% missingness rate in the full cohort).

### 3.5 Comparison of laboratory parameters and outcomes between breast and colorectal cancer patients

Colorectal cancer patients had significantly higher AST, white blood cell count, serum creatinine, and INR compared with breast cancer patients, and significantly lower serum albumin. ALT did not differ between the two groups, with identical medians observed in both. In-hospital mortality was substantially and significantly higher in colorectal cancer patients than in breast cancer patients. Breast cancer patients were younger and had better haemoglobin and lower creatinine, consistent with a less advanced systemic disease burden at the time of admission. The differences in creatinine and albumin between the two cancer groups are clinically meaningful: colorectal cancer is more likely to involve hepatic metastases and peritoneal disease, both of which impair albumin synthesis and increase renal load, whereas breast cancer patients in this cohort had predominantly non-metastatic disease at lower rates of visceral involvement (Table 5).

**TABLE 5:** Comparison of laboratory parameters and outcomes between breast and colorectal cancer patients.

Variable	Breast cancer Median [Q1–Q3] (n = 134)	Colorectal cancer Median [Q1–Q3] (n = 95)	p-value
Age (years)^a^	54.4 ± 11.7	60.9 ± 14.6	<0.001
Haemoglobin (g/dL)	12.1 [10.9–13.2]	10.9 [9.2–12.8]	0.002
WBC (×10^9^/L)	6.1 [4.7–8.8]	7.5 [5.2–10.9]	0.010
RBC (×10^6^/µL)	4.2 [3.8–4.7]	3.9 [3.4–4.5]	0.060
INR	1.1 [1.0–1.2]	1.2 [1.1–1.5]	<0.001
AST (U/L)	23.0 [16.0–31.0]	26.0 [19.0–49.5]	0.028
ALT (U/L)	21.0 [14.0–33.2]	21.0 [13.0–43.0]	0.944
Total protein (g/L)	69.0 [65.0–73.0]	64.0 [57.1–69.0]	<0.001
Serum albumin (g/L)	41.0 [37.0–43.0]	38.0 [29.0–42.0]	0.003

Data are presented as median [Q1–Q3] to reflect the interquartile range (25th and 75th percentiles) unless otherwise specified. Continuous non-normally distributed variables were compared using the Mann–Whitney U test.

^a^ Age data are presented as Mean ± SD and were analyzed using an independent samples t -test.

### 3.6 Drug-specific and cancer-specific comparisons

Electronic medical records were comprehensively reviewed to extract data on all prescribed medications, which were subsequently classified into non-oncological (supportive care), oncological (cancer-directed therapies), and other unclassified therapeutic agents. A total of 2,148 prescriptions were analyzed in the clinical cohort. Non-oncological supportive care medications represented the majority of the therapeutic burden, accounting for 59.03% ( n=1,268 ) of the total prescribed agents. This high proportion reflects the intensive supportive care management—including analgesics, antiemetics, and nutritional supplements—required alongside standard oncology regimens. Cancer-directed oncological therapies constituted 40.74% ( n=875 ) of the total prescriptions. Within the oncological drug classification, traditional cytotoxic chemotherapy was the predominant treatment strategy, representing 27.98% ( n=601 ) of overall prescriptions and 68.69% of all oncological agents. This was followed by targeted therapies at 6.84% ( n=147 ), hormonal therapies at 3.59% ( n=77 ), immunotherapies at 2.10% ( n=45 ), and radiotherapies at 0.23% ( n=5 ). A minor fraction of the dataset consisted of unmapped or miscellaneous placeholder entries, categorized as others/unspecified, which accounted for 0.23% ( n=5 ) of the aggregate prescription data (Table 6).

**TABLE 6:** Distribution and proportional breakdown of prescribed oncological and non-oncological medications across the clinical cohort (N = 2,148).

Drug classification	Treatment type/category	Total prescriptions	Overall proportion (%)
Non-oncological	Supportive care	1,268	59.03%
Oncological	Chemotherapy	601	27.98%
	Targeted therapy	147	6.84%
	Hormonal therapy	77	3.59%
	Immunotherapy	45	2.10%
	Radiotherapy	5	0.23%
Subtotal (oncological)	All cancer therapies	875	40.74%
Other	Others/unspecified	5	0.23%
Total	All prescriptions	2,148	100.00%

Data represent the absolute number and percentage distribution of prescriptions written across the entire patient cohort ( N=2,148 ). Percentages are calculated relative to the grand total of all categories combined.

#### 3.6.1 Drug-drug interaction profile

**FIGURE 3:**
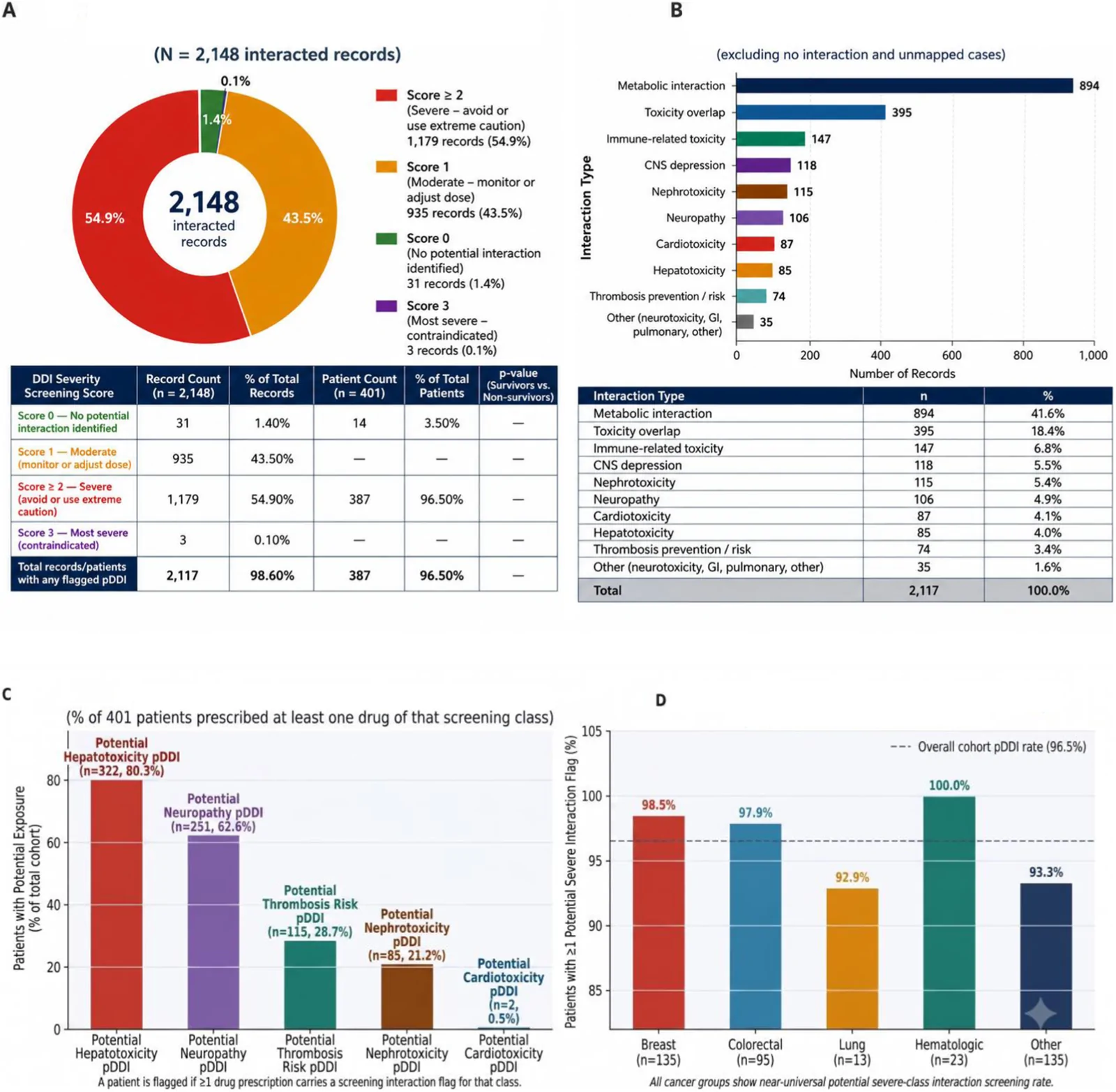
Drug-drug interaction profile–severity, type, organ exposure, and cancer group (n = 401 patients; 2,148 drug prescription records; source Drugs.com, accessed 2024–2026. Drug–Drug Interaction Profile Layout. **(A)** Percentage breakdown of total drug prescription records ( N=2,148 ) stratified by clinical severity tiers. **(B)** Absolute frequency and relative proportion distribution of underlying mechanical interaction classes. **(C)** Prevalence of individual organ-specific toxicity screening indicators across the patient cohort ( N=401 ). **(D)** Comparative penetration rates of severe clinical interaction vulnerabilities ( pDDI score≥2 ) cross-stratified against distinct baseline malignancy subgroups. Severity mapping adapted from the Drugs.com platform engine, integrating data vectors from Micromedex, Cerner Multum, and the American Society of Health-System Pharmacists (ASHP). All data counts verified against institutional tracking metrics.

A total of 2,148 drug prescriptions were evaluated across 401 patients, with a median of five drugs per patient [Q1 = 3, Q3 = 7]. Polypharmacy, defined as the concurrent use of five or more medications, was present in 206 patients (51.4%). Of the total prescription records, potential drug–drug interactions (pDDIs) were flagged in 2,117 instances (98.6%), leaving only 31 records (1.4%) completely devoid of identified interactions (Table 7; Figure 3A).

**TABLE 7:** DDI Severity Classification (per drug prescription record; N = 2,148 total records).

DDI severity screening score	Record count (n = 2,148)	% of total records	Patient count (n = 401)	% of total patients
Score 0 — No potential interaction identified	31	1.40%	14	3.50%
Score 1 — moderate (monitor or adjust dose)	935	43.50%	—	—
Score ≥2 — severe (avoid or use extreme caution)	1,179	54.90%	387	96.50%
Score 3 — most severe (contraindicated)	3	0.10%	—	—
Total records/patients with any flagged pDDI	2,117	98.60%	387	96.50%

pDDI, potential drug-drug interaction. Percentages may not total exactly 100% due to rounding. Patient count totals omit scores where multiple interactions per patient span different categories, representing baseline distribution.

Among these flagged interactions, severe risk profiles predominated: 54.9% ( n=1,179 ) were categorized as severe (severity score = 2) and 0.1% ( n=3 ) carried the most severe, contraindicated classification (severity score = 3). Moderate risks (severity score = 1) accounted for 43.5% ( n=935 ). Specific named pDDI pairs, where the screening tool identified a distinct interacting partner by name, were recorded in 529 instances; of these, 503 (95.1%) fell within the severe classification tier. At the patient level, 387 out of 401 individuals (96.5%) were prescribed at least one medication carrying a screening flag for a severe potential interaction (Table 7).

Stratification by mechanistic classification (Table 8; Figure 3B) revealed that potential metabolic interactions were the most frequent tier (894 records, 41.6%), reflecting predicted cytochrome P450 (CYP)-mediated competition between antineoplastic agents and co-prescribed antimicrobials, corticosteroids, or antiemetics. Potential toxicity overlap was the second most frequent category (395 records, 18.4%), followed by screening flags for potential immune-related toxicity (147 records, 6.8%), potential central nervous system (CNS) depression (118 records, 5.5%), potential nephrotoxicity (115 records, 5.4%), and potential neuropathy (106 records, 4.9%).

**TABLE 8:** Interaction type distribution (2,148 records).

Interaction type	n	%
Metabolic interaction	*894*	*41.6%*
Toxicity overlap	395	18.4%
Immune-related toxicity	147	6.8%
CNS depression	118	5.5%
Nephrotoxicity	115	5.4%
Neuropathy	106	4.9%
Cardiotoxicity	87	4.1%
Hepatotoxicity	85	4.0%
Thrombosis prevention/risk	74	3.4%
Other (neurotoxicity, GI, pulmonary, other)	35	1.6%

#### 3.6.2 Statistical evaluation of organ-specific toxicity exposures and subgroup prevalence

To assess whether the pharmacological profile of prescribed regimens was statistically associated with clinical outcomes, exposure to medications carrying screening flags for potential organ-specific toxicities was evaluated by survival cohort (Table 9). Potential hepatotoxic medication exposure was highly prevalent but did not vary significantly between groups (82.7% of survivors vs. 73.3% of non-survivors; p=0.056 ). Notably, potential neuropathic medication exposure was significantly more prevalent among survivors than non-survivors (66.7% vs. 50.5%; p=0.005 ). Rather than reflecting a pharmacologically protective effect, this highlights a survivorship pattern: taxane-based regimens, which drive these neuropathic flags, were disproportionately administered to stable patients who completed treatment, whereas critically ill patients were less likely to receive full cytotoxic cycles before clinical deterioration. Exposure to medications with potential thrombosis risks (29.0% vs. 27.7%; p=0.906 ) and nephrotoxic drug exposure (20.3% vs. 23.8%; p=0.556 ) did not differ significantly between groups, while potential cardiotoxic exposure remained rare across the entire cohort (0.5% overall; p=1.000 ).

**TABLE 9:** Organ-Specific Drug Exposure by Discharge Outcome (patient level).

Organ category	n patients (%)	Alive n (%)	Dead n (%)
Hepatotoxic drug exposure	322 (80.3%)	248 (82.7%)	74 (73.3%) p = 0.056
Neuropathic drug exposure	251 (62.6%)	200 (66.7%)	51 (50.5%) p = 0.005 *
Thrombosis-risk drug exposure	115 (28.7%)	87 (29.0%)	28 (27.7%) p = 0.906
Nephrotoxic drug exposure	85 (21.2%)	61 (20.3%)	24 (23.8%) p = 0.556
Cardiotoxic drug exposure	2 (0.5%)	1 (0.3%)	1 (1.0%) p = 1.000

* Indicates statistical significance at p < 0.05.

Collectively, these screening data indicate that pre-existing patient-specific organ dysfunction—such as baseline renal disease ( p<0.001 )—rather than database-flagged interaction categories *per se*, acts as the dominant determinant of mortality risk. Descriptively, patients with potential nephrotoxic medication exposure in the absence of renal disease had a mortality rate of 23.5% (16 of 68), tracking closely with the 21.1% mortality rate (59 of 279) of those with neither risk factor. Conversely, patients presenting with pre-existing renal disease alone experienced a mortality rate of 48.6% (18 of 37), which converged with the 47.1% mortality rate (8 of 17) observed in patients with both risk factors, underscoring baseline renal impairment as the primary risk driver.

Patient-level screening for organ-specific potential toxicities (Figure 3C) confirmed that potential hepatotoxicity flags were the most widespread (80.3%), followed by flags for potential neuropathy (62.6%), potential thrombosis risks (28.7%), potential nephrotoxicity (21.2%), and potential cardiotoxicity (0.5%).

When evaluating severe interactions across specific oncological classifications (Table 10; Figure 3D), near-universal exposure to at least one high-risk flag ( pDDI score≥2 ) was observed across all cohorts, yielding an overall patient-level baseline penetration rate of 96.5% ( n=387 ). The highest risk profile was observed in the hematologic malignancy group, which demonstrated an absolute 100.0% penetration rate ( n=23 ). The severe interaction rates remained profoundly elevated in both the breast cancer and colorectal cancer subgroups at 98.50% ( n=133/135 ) and 97.90% ( n=93/95 ), respectively. The lowest comparative rates were found in the lung cancer subgroup (92.90%) and the heterogeneous “Other” malignancy classification (93.30%), though both subsets still closely mirrored the pervasive interaction burden observed across the wider study population.

**TABLE 10:** Stratification of potential organ-specific toxicities and patient-level high-risk interaction rates by malignancy category.

Cancer group	Patient subgroup (n)	Patients with ≥1 potential severe interaction flag (n)^a^	Subgroup severe pDDI rate (%)	Reference cohort rate (%)
Breast	135	133	98.50%	96.50%
Colorectal	95	93	97.90%	96.50%
Lung	13	12	92.90%	96.50%
Hematologic	23	23	100.00%	96.50%
Other	135	126	93.30%	96.50%
Overall cohort	401	387	96.50%	—

pDDI = potential drug–drug interaction. a ‐ Potential severe drug–drug interactions were defined as major, contraindicated, or high-risk interactions requiring clinical intervention, intensive monitoring, or alternative therapy, identified using standard interaction screening software.

#### 3.6.3 Comparative profile of paclitaxel and oxaliplatin treatment sub-cohorts

Among patients receiving paclitaxel-based therapy ( n=85 ) and those receiving oxaliplatin-based regimens ( n=81 ), no significant differences were found in baseline liver enzymes (AST, ALT), serum albumin, white blood cell count (WBC), or overall length of hospital stay. However, serum creatinine was significantly lower in the paclitaxel cohort compared to the oxaliplatin cohort ( 61 μmol/L [52–75] vs. 72 μmol/L [66–91], p=0.007 ), reflecting the known nephrotoxic profile and platinum-class effect associated with oxaliplatin. The international normalized ratio (INR) was also significantly lower in the paclitaxel group ( 1.1 [1.1–1.2] vs. 1.2 [1.1–1.6], p=0.017 ), suggesting a greater underlying coagulopathy burden in patients receiving oxaliplatin, which may be secondary to a higher degree of hepatic involvement or tumor burden in their primary colorectal or gastric malignancies. Overall, in-hospital outcomes did not differ significantly between the two therapeutic groups (Table 11).

**TABLE 11:** Comparison of laboratory parameters and outcomes between paclitaxel and oxaliplatin.

Variable	Paclitaxel median [Q1, Q3](n = 85)	Oxaliplatin median [Q1, Q3](n = 81)	p-value
AST (U/L)	25 [17, 44]	24 [19, 41]	0.874
ALT (U/L)	25 [16, 37]	20 [14, 27]	0.247
Albumin (g/L)	39 [34, 43]	38 [28, 43]	0.383
Creatinine (μmol/L)	61 [52, 75]	72 [66, 91]	0.007
WBC (×109/L)	6.4 [4.7, 10.4]	7.6 [6.0, 10.5]	0.167
INR	1.1 [1.1, 1.2]	1.2 [1.1, 1.6]	0.017
Hospital stay (days)	1 [0, 4]	1 [0, 5]	0.284

Data are presented as Median [Q1, Q3]. p -values were calculated using the Mann–Whitney U test for continuous variables. A p -value <0.05 indicates statistical significance.

### 3.7 Potential drug–drug interaction profile

Across the final analytical cohort ( N=401 patients) with a total of 2,148 active inpatient prescriptions evaluated, potential drug–drug interactions (pDDIs) were extensively characterized across mechanistic, severity, organ-specific, and oncological domains (Table 12). Mechanistically, *Pharmacokinetic Pathways* driven by metabolic interactions (e.g., Cytochrome P450 [ CYP3A4 ] pathways) represented the primary driver of interactions, accounting for 41.6% ( n=894 ) of the total identified pDDIs, while *Pharmacodynamic Pathways* characterized by toxicity overlaps constituted a substantial secondary driver, comprising general toxicity overlap ( 18.4% ; n=395 ), nephrotoxicity ( 5.4% ; n=115 ), neuropathy ( 4.9% ; n=106 ), and cardiotoxicity ( 4.1% , n=87 ). When evaluated by clinical severity per individual interaction, the vast majority were classified as severe (score ≥2 ), comprising 85.8% ( n=344 ) of the profile, followed by moderate interactions (score 1) at 11.0% ( n=44 ) and minor interactions (score 0) at 3.2% ( n=13 ). Regarding specific antineoplastic protocols, paclitaxel (combined regimens) was the most frequent driver of pDDIs, accounting for 6.5% ( n=26 ) of the cohort’s profile, followed closely by the doxorubicin/AC protocol ( 6.2% , n=25 ), docetaxel ( 3.2% , n=13 ), oxaliplatin ( 1.7% , n=7 ), and irinotecan ( 1.5% , n=6 ). At the individual patient level ( N=401 ), organ-specific exposures carrying DDI risks were highly prevalent; a total of 83.5% of patients ( n=335 ) were exposed to at least one hepatotoxic DDI during their stay, while 62.6% ( n=251 ) experienced an exposure carrying a risk of induced neuropathy, 30.4% ( n=122 ) were exposed to a thrombosis-risk interaction, and 21.2% ( n=85 ) faced a nephrotoxic DDI exposure. Finally, stratification of severe pDDIs across primary tumor classifications revealed distinct variations among the clinical subgroups, with the highest prevalence of severe interactions observed within the colorectal cancer subgroup ( 91.6% , n=87 of 95 ), followed by the gynecologic cancer group ( 88.5% , n=23 of 26 ) and the breast cancer cohort ( 83.7% , n=113 of 135 ) (Table 12).

**TABLE 12:** Drug–drug interaction profile of the study cohort (N = 401, total drugs in prescriptions N = 2,148).

Domain	Category	n	%
Most frequent drugs in DDIs	Paclitaxel (combined)	26	6.5
	Docetaxel	13	3.2
	Doxorubicin/AC protocol	25	6.2
	Oxaliplatin	7	1.7
	Irinotecan	6	1.5
Mechanistic classification (per interaction)			
Pharmacokinetic pathways	Metabolic interactions (e.g., CYP3A4 pathways)	894	41.6
Pharmacodynamic pathways	General toxicity overlap	395	18.4
	Nephrotoxicity	115	5.4
	Cardiotoxicity	87	4.1
	Neuropathy	106	4.9
DDI severity (per interaction)	Severe (score ≥2)	344	85.8
	Moderate (score 1)	44	11.0
	Minor (score 0)	13	3.2
Organ-specific DDI exposure (per patient)	Any hepatotoxic DDI	335	83.5
	Any nephrotoxic DDI	85	21.2
	Any neuropathy DDI	251	62.6
	Any thrombosis-risk DDI	122	30.4
Severe DDIs by cancer group	Breast cancer (n = 135)	113	83.7
	Colorectal cancer (n = 95)	87	91.6
	Gynecologic cancer (n = 26)	23	88.5

### 3.8 Correlation between clinical parameters and in-hospital mortality

**FIGURE 4:**
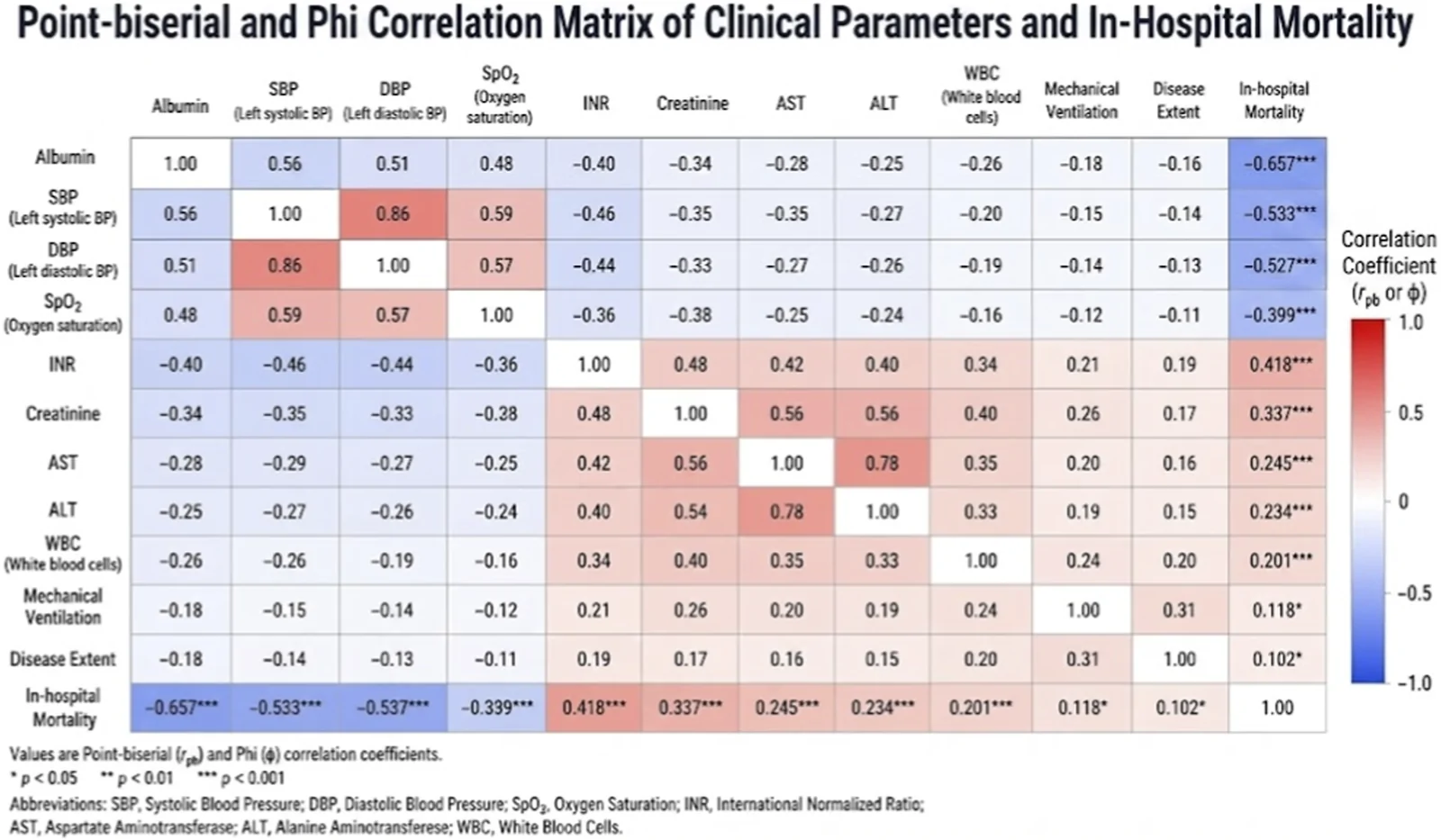
Point-biserial and phi correlation matrix of clinical parameters and in-hospital mortality.

Correlation analyses demonstrated statistically significant relationships with in-hospital mortality (all p<0.05 ; Table 13 and Figure 4). Moderate-to-strong negative correlations were found for baseline albumin ( $r_{pb}=-0.657,p<0.001$ ), left systolic blood pressure ( $r_{pb}=-0.533,p<0.001$ ), and left diastolic blood pressure ( $r_{pb}=-0.527,p<0.001$ ), alongside a moderate negative correlation with oxygen saturation ( $r_{pb}=-0.399,p<0.001$ ). Conversely, positive correlations tracked with increased mortality risk: moderate relationships were noted for international normalized ratio (INR; $r_{pb}=0.418,p<0.001$ ) and serum creatinine ( $r_{pb}=0.337,p<0.001$ ), while weak-to-moderate positive associations were identified for serum aspartate aminotransferase (AST; $r_{pb}=0.245,p<0.001$ ), alanine aminotransferase (ALT; $r_{pb}=0.234,p<0.001$ ), white blood cell count ( $r_{pb}=0.201,p<0.001$ ), mechanical ventilation requirements ( ϕ=0.118,p=0.018 ), and radiological disease extent ( $r_{pb}=0.102,p=0.042$ ).

**TABLE 13:** Point-biserial and phi correlation coefficients between clinical parameters and in-hospital mortality.

Variable	Variable type	Appropriate statistical method	Correlation/metric (rpb or ϕ)	Interpretation	p-value
Albumin	Continuous	Point-biserial (rpb)	−0.657	Moderate-to-strong negative: higher baseline albumin is strongly associated with survivorship (protective factor)	<0.001
Left systolic BP	Continuous	Point-biserial (rpb)	−0.533	Moderate-to-strong negative: higher systolic blood pressure is associated with survivorship (hypotension tracks with mortality)	<0.001
Left diastolic BP	Continuous	Point-biserial (rpb)	−0.527	Moderate-to-strong negative: higher diastolic blood pressure is associated with survivorship (hypotension tracks with mortality)	<0.001
Oxygen saturation	Continuous	Point-biserial (rpb)	−0.399	Moderate negative: lower baseline SpO2 is significantly associated with increased mortality	<0.001
INR	Continuous	Point-biserial (rpb)	0.418	Moderate positive: coagulopathy (higher INR) is moderately associated with increased mortality risk	<0.001
Creatinine	Continuous	Point-biserial (rpb)	0.337	Moderate positive: renal impairment (higher serum creatinine) is moderately associated with increased mortality risk	<0.001
AST	Continuous	Point-biserial (rpb)	0.245	Weak-to-moderate positive: hepatic injury indicator (elevated AST) tracks with higher mortality	<0.001
ALT	Continuous	Point-biserial (rpb​)	0.234	Weak-to-moderate positive: hepatic injury indicator (elevated ALT) tracks with higher mortality	<0.001
WBC count	Continuous	Point-biserial (rpb)	0.201	Weak-to-moderate positive: leukocytosis (systemic inflammation/infection) tracks with higher mortality	<0.001
Mechanical ventilation	Binary (0/1)	Phi coefficient (ϕ)/Chi-square	0.118	Weak positive: requirement for mechanical ventilation is associated with increased mortality	0.018
Disease extent	Ordinal/continuous	Point-biserial (rpb) or spearman ρ	0.102	Weak positive: greater radiological extent of disease weakly tracks with increased mortality	0.042

In-hospital mortality is recorded as a binary outcome ( 0=Survivor ; 1=Deceased ). Bivariate correlations between clinical parameters and mortality were calculated using point-biserial correlation ( $r_{pb}$ ) for continuous metrics and the phi coefficient ( ϕ ) for dichotomous categorical metrics. Both yield numerical values mathematically equivalent to Pearson’s r under binary coding.

### 3.9 Multivariable logistic regression analysis

**FIGURE 5:**
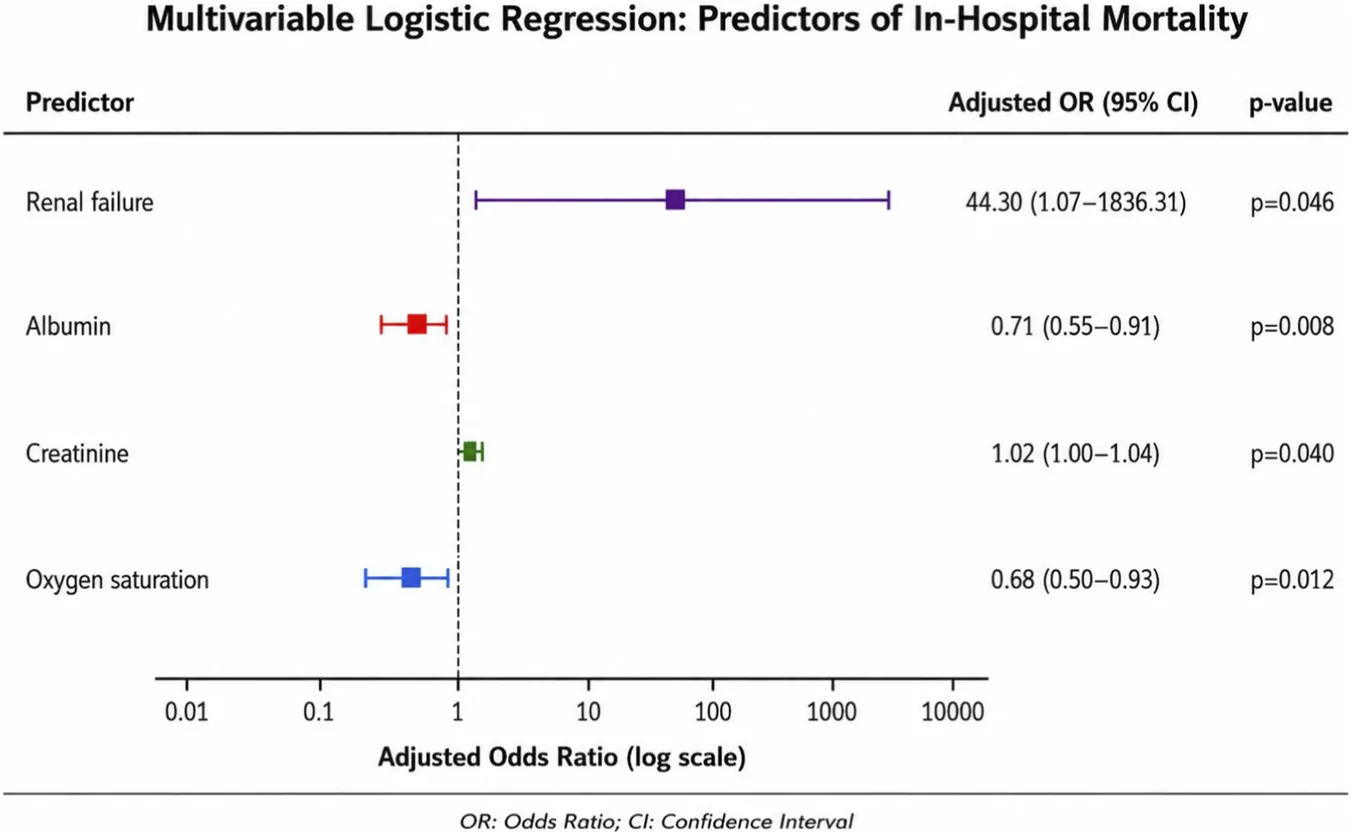
Forest plot displaying Adjusted Odds Ratios ( aOR ) and 95% Confidence Intervals ( CI ) on a logarithmic scale for independent physiological predictors of in-hospital mortality. The vertical dashed line at aOR=1.0 represents no effect. Factors positioned to the right ( aOR>1.0 ) indicate increased mortality risk, whereas factors to the left ( aOR<1.0 ) indicate protective factors associated with survivorship. Mechanical ventilation was excluded from the multivariable plot due to high collinearity with acute organ dysfunction markers.

Multivariable binary logistic regression identified four independent physiological predictors of in-hospital mortality: renal failure, baseline serum albumin, serum creatinine, and oxygen saturation. The multivariable model demonstrated exceptional discrimination and overall classification performance ( AUC=0.953 ; Table 14). Overall in-hospital mortality in this cohort reflects a complex interplay between underlying oncological disease severity and acute physiological decompensation. Across non-survivors, hemodynamic instability, anemia, acute renal dysfunction, and systemic inflammatory activation followed a consistent, predictable trend. Taken together, these findings indicate that overall physiological maladaptation and multi-organ strain—rather than isolated laboratory aberrations or database-flagged drug interactions alone—characterize the patients at the highest risk of in-hospital mortality (Table 14; Figures 4, 5).

**TABLE 14:** Multivariable logistic regression analysis for predictors of in-hospital mortality.

Variable	B	SE	Wald	Adjusted OR	p value
Renal failure	3.79	1.90	3.99	44.29	0.046
Albumin	−0.35	0.13	7.14	0.71	0.008
Creatinine	0.02	0.01	4.22	1.02	0.040
Oxygen saturation	−0.39	0.16	6.25	0.68	0.012

Treatment interventions (e.g., mechanical ventilation) and highly collinear redundant markers were excluded from the final multivariable binary logistic regression model to prevent statistical suppression, overadjustment, and severe multicollinearity with primary organ dysfunction variables (e.g., renal failure and low ${\text{SpO}}_{2}$ ).

### 3.10 Polypharmacy, interaction effects, and mortality

Polypharmacy was evident in about half of the cohort, and it did not solely correlate with mortality. But drug-drug interaction effects showed a statistically significant association with mortality. Moreover, even though the level of polypharmacy was very high, it was not associated with mortality when it was adjusted with confounders. This observation suggests that the only number of drugs might not be significant but the clinical circumstances surrounding their prescription Table 15.

**TABLE 15:** Polypharmacy status, association with in-hospital mortality, and regression analysis.

Polypharmacy/Variable	n (%)	Alive n	Dead n	OR	p value
Polypharmacy status					
No	195 (48.6)	141	54	—	—
Yes	206 (51.4)	160	46	0.762	0.244
Interaction-related variables					
Interaction effect	—	—	—	0.994	0.015
Interaction type	—	—	—	1.012	0.597

Dashes (—) indicate variables or cells for which the measure was not applicable or not estimated in the corresponding analysis (e.g., descriptive or univariate measures not included in regression models.

### 3.11 Receiver operating characteristics analysis

**FIGURE 6:**
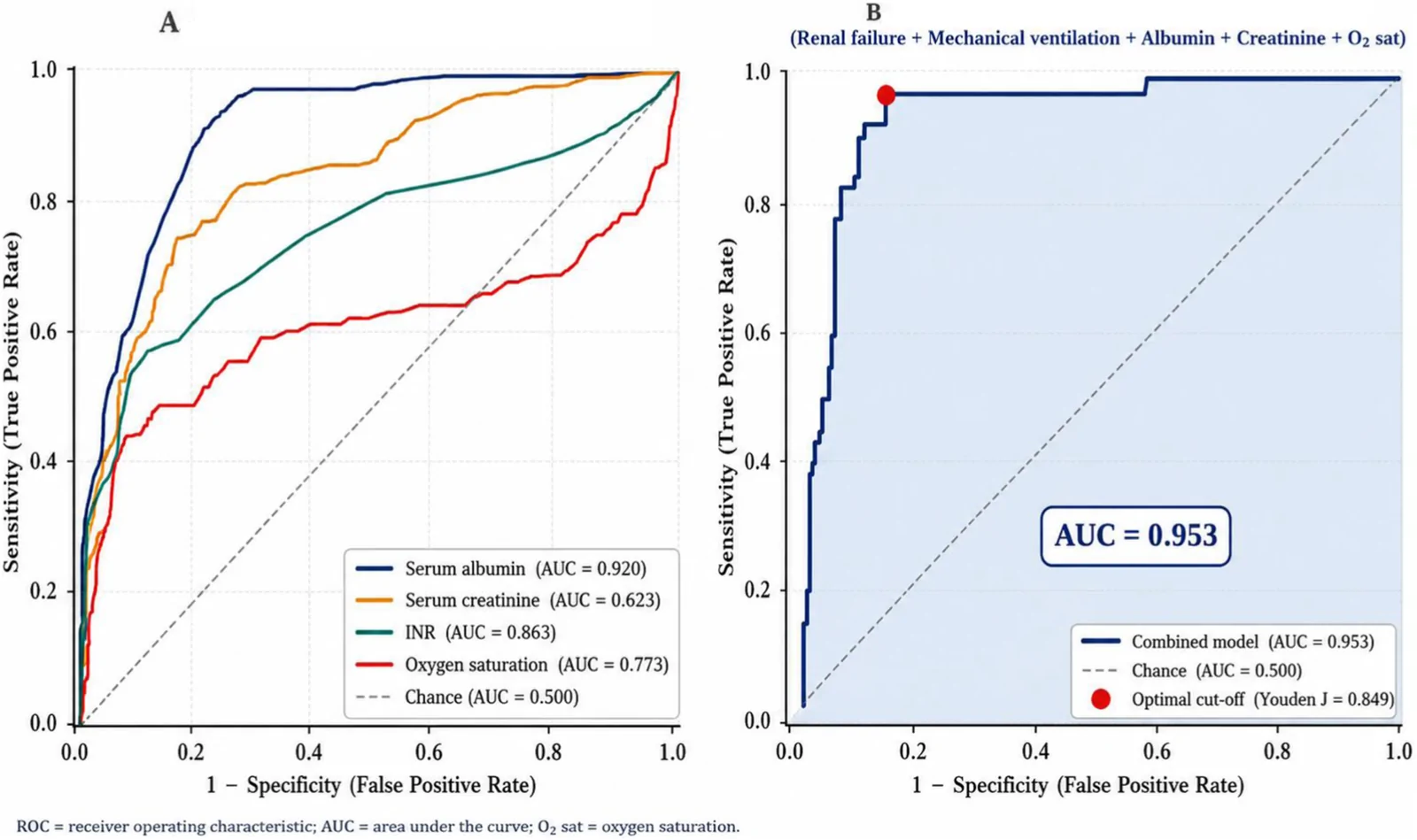
**(A)** Individual ROC curves for key biomarkers. **(B)** Multivariable ROC curve combining clinical predictors and biomarkers (AUC = 0.953). The optimal decision threshold is indicated by the red marker (Youden J = 0.849). Model performance: Nagelkerke R^2^ = 0.71; Hosmer–Lemeshow test p = 0.14. Abbreviations: AUC, area under the curve; ROC, receiver operating characteristic; O_2_ Sat, oxygen saturation.

ROC curve analysis confirmed serum albumin as the strongest single predictor of in-hospital mortality, with an AUC of 0.920 . At the Youden-optimal cut-off of 35 g/L , albumin yielded a sensitivity of 98.0% and specificity of 75.8% , consistent with the established clinical threshold for hypoalbuminemia. INR was the second most discriminating individual variable ( AUC=0.863 ): at an optimal cut-off of 1.28 , sensitivity was 75.2% and specificity was 85.9% , reflecting the clinical utility of coagulopathy as a marker of hepatic dysfunction and systemic failure. Oxygen saturation demonstrated fair discrimination ( AUC=0.773 ); its Youden-optimal cut-off of 92% yielded a sensitivity of 57.6% and specificity of 90.9% . This estimate should be interpreted with caution given the 22.4% overall missingness in oxygen saturation data, which was concentrated among acutely unwell patients admitted in extremis. Serum creatinine showed more modest individual discrimination ( AUC=0.623 ): at the optimal cut-off of 116 μmol/L , specificity was high at 94.6% , but sensitivity was limited to 45.0% . This indicates that while marked creatinine elevation ( ≥116 μmol/L ) is highly specific for mortality risk, a significant proportion of non-survivors succumb without reaching extreme renal biomarker thresholds. Individual ROC curves are displayed in Figures 6A,B.

The combined multivariable logistic regression model—incorporating renal failure, serum albumin, serum creatinine, and oxygen saturation—achieved an overall AUC of 0.953 ( 95% CI:0.931−0.975 ) in patients with complete data across all predictor variables. The Youden-optimal operating point identified a sensitivity of 98.3% and specificity of 86.6% , corresponding to a Youden J statistic of 0.849 . This level of discrimination substantially exceeds that of any individual biomarker alone and aligns with published combined risk prediction models in hospitalized oncology populations, where ensemble approaches routinely report AUC values between 0.80 and 0.95 (Yuan and Kabir, 2025). The combined model AUC reflects apparent rather than cross-validated performance, as the model was derived and evaluated in the same dataset. External validation in a prospective multicenter cohort is required before clinical use.

### 3.12 Summary of key results

In-hospital mortality within this cohort was primarily driven by acute physiological derangements and multi-organ dysfunction rather than baseline demographics or specific oncological diagnoses. The strongest, most consistent independent predictors of in-hospital mortality included acute renal dysfunction (manifested by elevated serum creatinine and clinical renal failure), severe hypoalbuminemia, and hypoxemia (reduced oxygen saturation), supported by unadjusted bivariate associations with hemodynamic instability and mechanical ventilation requirements. Conversely, baseline demographic factors and primary cancer types alone did not significantly impact discharge status, underscoring that short-term mortality is dictated by acute physiological decline rather than underlying malignant classification.

## 4 Discussion

The clinical management of patients undergoing oncological treatment involves a delicate balance between administering highly potent antineoplastic regimens and executing robust supportive care to mitigate systemic toxicities and tumor-related symptoms. Analysis of the 2,148 prescriptions within this cohort reveals that non-oncological supportive medications comprised the clear majority of the therapeutic burden (59.03%, n=1,268 ), emphasizing the intensive polypharmacy environment required to sustain oncology patients through active treatment. Conversely, cancer-directed therapies accounted for 40.74% ( n=875 ) of the total prescriptions, with traditional cytotoxic chemotherapy serving as the predominant treatment strategy (27.98% of overall prescriptions). This extensive multi-drug landscape underscores an unavoidable exposure to complex drug-drug interactions (DDIs), which demand precise risk stratification to protect patient safety without compromising oncological efficacy. Crucially, the demographic features and cancer type alone were not the main factors contributing to in-hospital deaths in this retrospective study. Instead, acute physiological derangements and multi-organ dysfunction predominated in driving the mortality rate of this multinational oncology cohort treated in a tertiary care centre in Saudi Arabia. The most powerful independent predictors of in-hospital mortality proved to be renal failure, hypoalbuminemia, elevated serum creatinine, and hypoxemia (reduced oxygen saturation), complemented by significant unadjusted associations with hemodynamic instability and the necessity for mechanical ventilation. These findings highlight that systemic clinical decompensation and acute illness severity—rather than baseline cancer characteristics or database-flagged interactions alone—are the focal determinants of short-term outcomes in hospitalized cancer patients (Zhu et al., 2021; Huang et al., 2022).

Among these, renal dysfunction emerged as an exceptionally powerful independent predictor of mortality. Loss of renal function serves as a critical marker of both pre-existing comorbidity and treatment-induced pathology. For example, platinum-based compounds are highly nephrotoxic and frequently induce acute tubular damage, cellular necrosis, and severe electrolyte imbalances. Furthermore, kidney dysfunction fundamentally alters the pharmacokinetics and clearance of antineoplastic agents, consequently exposing the system to a greater free fraction of cytotoxic substances and compounding systemic toxicity. This bidirectional, hazardous interaction between deteriorating renal function and targeted pharmacotherapy underscores its strong correlation with increased in-hospital mortality across multiple malignancies, including lung, breast, and colorectal cancers (Kitchlu et al., 2022; Wang et al., 2025).

Similarly, hypoalbuminemia demonstrated a significant independent relationship with in-hospital mortality. As a classic surrogate marker of malnutrition, catabolic stress, and chronic systemic inflammation, reduced serum albumin concentrations fundamentally alter drug binding kinetics (Chien et al., 2023; Di Castelnuovo et al., 2024). A drop in albumin increases the circulating free drug fraction, leading to unpredictable pharmacokinetic shifts that predispose patients to severe, unmanaged toxicities. This underscores the predictive utility of routine nutritional and biochemical profiling, reinforcing the necessity of preemptive nutritional optimization in acute care settings.

Indicators of hemodynamic and physical instability, such as hypotension, hypoxemia, and elevated inflammatory markers, were also tightly coupled with adverse outcomes (Liu et al., 2022). While mechanical ventilation was a dominant predictor of mortality, its clinical value should be interpreted as an indicator of critical illness severity rather than an isolated therapeutic risk factor. Advanced respiratory failure, fulminant sepsis, and multiorgan breakdown characterize the patients requiring ventilatory support in this cohort (Haviland et al., 2020). While polypharmacy was highly prevalent due to local prescriptive trends and a heavy baseline burden of infections, it was not an independent predictor of death on its own. Instead, the net presence of severe drug-drug interactions underpinned the statistical correlation with mortality, bringing medication safety to the forefront of oncology care (Le et al., 2024; Mok et al., 2025).

Beyond traditional drug-drug interactions, underlying pharmacogenetic variability plays a critical role in modulating systemic drug exposure and idiosyncratic toxicity profiles within this cohort. For instance, polymorphisms in the *DPYD* gene, which encodes the rate-limiting enzyme dihydropyrimidine dehydrogenase, severely impair the catabolism of fluorouracil (5-FU), predisposing carriers to life-threatening myelosuppression and severe mucositis even under standard dosing regimens (Amstutz et al., 2018). Similarly, genetic variations in *UGT1A1* (such as the UGT1A1*28 allele) reduce the glucuronidation of SN-38, the active metabolite of irinotecan, driving up the risk of severe, dose-limiting neutropenia and debilitating diarrhea (Caudle et al., 2014).

This risk landscape is further complicated by the widespread, often unguided, concomitant use of natural products and herbal supplements among oncology patients. Many common botanical supplements function as potent modulators of cytochrome P450 pathways (such as CYP3A4 inhibition or induction) or drug transporters like P-glycoprotein (Fasinu and Rapp, 2019). When taken alongside narrow-therapeutic-index agents such as paclitaxel or targeted tyrosine kinase inhibitors, these natural products can either precipitously drop systemic drug levels—leading to therapeutic failure—or dangerously elevate them, exacerbating organ-specific toxicities like the hepatotoxicity and nephrotoxicity tracked in this study (Goey et al., 2014).

Collectively, the clinical implications of these overlapping variables emphasize the urgent transition toward personalized medicine in oncology. Retrospective stratification of clinical DDIs alone is no longer sufficient; true optimization of patient care requires a proactive framework. Integrating pre-therapeutic companion diagnostic screening—such as targeted *DPYD* and *UGT1A1* genotyping—alongside comprehensive, pharmacist-led reconciliation of both prescriptive medications and over-the-counter herbal supplements is essential (Relling and Klein, 2011). By customizing drug selection and preemptively adjusting dosages based on an individual’s unique genomic and polypharmacy profile, clinical teams can significantly mitigate the severe interaction risks (Scores ≥ 2) identified in this cohort, ultimately safeguarding therapeutic efficacy while minimizing preventable in-hospital morbidity and mortality.

In unadjusted univariate analyses, advanced age was significantly linked to mortality, a finding consistent with decreased physiological reserve, multimorbidity, and heightened vulnerability to aggressive oncologic regimens. Although age was not retained in the final multivariable model, its prognostic impact is likely mediated through age-related organ frailty and functional decline (Gopishetty et al., 2020; Extermann et al., 2021; Kahn et al., 2022). Similarly, male sex was associated with higher mortality rates in univariate models, mirroring broader cancer epidemiology trends that are often driven by differences in comorbidity loads, delayed healthcare-seeking behaviors, and disease severity at initial presentation rather than immutable biological differences (Degerli et al., 2024; Qian et al., 2024).

Notably, smoking status was significantly related to an increased risk of in-hospital mortality, reinforcing extensive data showing that active smoking impairs immune responses, augments cardiopulmonary complications, and blunts the efficacy of antineoplastic therapies (Malyszko et al., 2020; Moradi et al., 2024). Conversely, mounting evidence demonstrates that smoking cessation following a cancer diagnosis yields substantially better prognosis and discharge outcomes across various malignancies, particularly lung and head and neck tumors (Caini et al., 2022; Wang et al., 2023). This strong association highlights the profound clinical significance of implementing structured, compulsory smoking cessation interventions within oncology care, particularly at the time of acute hospital admission when patient engagement with the healthcare infrastructure is maximally enhanced (Koczwara et al., 2023; Sheikh and Evison, 2025).

The choice of Drugs.com as the primary DDI screening platform warrants direct comment in the context of published performance data. The platform integrates data from Micromedex, Cerner Multum, and the American Society of Health-System Pharmacists (ASHP), efficiently providing a composite output from three independent, validated sources through a single query interface. In a head-to-head comparison of nine DDI screening tools evaluating oral oncology drug pairs, Drugs.com achieved the highest performance accuracy score among freely available tools (0.83), demonstrating no statistically significant difference from the top-performing subscription database, Lexicomp® (0.89) (Marcath et al., 2018).

A subsequent evaluation across 229 oral antineoplastic drug pairs confirmed that Drugs.com (accuracy score 352 of 400) and Lexicomp® (355 of 400) both substantially outperformed other major tools, including Micromedex (270 of 400) and Medscape (280 of 400) (Bossaer et al., 2022). These established benchmarks support its application as a highly credible, accurate screening platform in retrospective oncology research settings where access to cost-prohibitive subscription software may be restricted. For pharmacokinetic interactions—such as CYP3A4 metabolic alterations driven by paclitaxel and docetaxel regimens—simple administration-time separation is ineffective due to the prolonged, systemic modulation of hepatic enzyme activity (Scripture and Figg, 2006). Management requires proactive dose modifications of narrow-therapeutic-index supportive care agents or transitioning to non-interacting alternatives (Ramos-Esquivel et al., 2017). Conversely, pharmacodynamic pDDIs characterized by cumulative systemic overlaps demand vigilant clinical and laboratory surveillance rather than scheduling adjustments. Specifically, these toxicities necessitate regular complete blood count monitoring to mitigate overlapping myelosuppression, routine serum creatinine tracking to manage the 21.2% patient-level nephrotoxic exposure rate, and serial electrocardiographic tracking to actively screen for additive corrected QT ( QTc ) interval prolongation (Lyon et al., 2022).

Several limitations of the present study must be acknowledged. First, the primary outcome measure was restricted to in-hospital mortality. Valuable intermediate and longitudinal outcomes, such as intensive care unit (ICU) mortality metrics, post-discharge mortality proportions, and exact length of stay parameters, were not evaluated and remain important avenues for future prospective studies. Second, the retrospective, single-center design naturally limits direct causal inferences and immediate geographic generalizability, as findings may partially reflect localized prescription trends, specific supportive care capabilities, or regional infection burdens. Furthermore, the absence of consistently documented functional performance status scores (e.g., ECOG or Karnofsky) and definitive treatment intent (curative vs. palliative) creates a possibility for residual confounding.

Additionally, while the sample size of 401 patients was sufficient for the primary multivariable analysis, it limited the statistical power required for robust subgroup comparisons. The combined model’s excellent area under the receiver operating characteristic curve (AUC = 0.953) represents an apparent rather than cross-validated discrimination metric, as the model was derived and evaluated within the same complete-case dataset ( n=305 patients with complete data across all five primary predictors). Finally, interactions were classified using a single DDI database and were not independently cross-referenced against a secondary database at the exact point of data collection. Future prospective multi-center studies utilizing dual-database verification protocols are recommended to further strengthen drug interaction severity classifications and enhance the generalizability of these findings.

## 5 Conclusion

The findings of this retrospective study demonstrate that short-term, in-hospital mortality among acute oncology inpatients is primarily driven by acute physiological derangements and organ failure rather than baseline oncological characteristics or demographic variables. Specifically, renal failure ( aOR=44.29 ), baseline hypoalbuminemia ( aOR=0.71 ), elevated serum creatinine ( aOR=1.02 ), and reduced oxygen saturation ( aOR=0.68 ) were identified as robust, independent clinical predictors of in-hospital mortality. Among these, deterioration of the renal axis emerged as the most formidable prognostic indicator. Impaired kidney function not only marks systemic critical illness but directly compromises the clearance of narrow-therapeutic-index antineoplastic agents, exposing patients to prolonged cytotoxic drug exposure and exacerbating systemic treatment-related toxicity. Concurrently, profound hypoalbuminemia serves as a key surrogate for severe catabolic stress and systemic inflammation, fundamentally altering drug–protein binding kinetics and increasing the circulating free fraction of active drugs to dangerous thresholds.

From a pharmacological safety standpoint, this multinational cohort exposed a critical clinical vulnerability: a near-universal exposure to severe-class drug-drug interactions (DDIs), with 97.5% of the active patient population experiencing at least one severe (Score ≥ 2) interaction risk. Crucially, multi-variable modeling demonstrated that simple polypharmacy—defined merely by the sheer quantity of concurrent medications—does not independently predict in-hospital death. Instead, it is the qualitative composition of the drug combinations rather than the absolute drug count that constitutes the actual interaction-related clinical risk factor. Cornerstone oncology protocols, such as 5-fluorouracil (5-FU), oxaliplatin, and irinotecan regimens (e.g., FOLFOX, FOLFIRI, XELOX/CapOx), are frequently combined with intensive supportive care agents (analgesics, antiemetics, and proton-pump inhibitors), creating highly intricate metabolic competition and profound toxicity overlaps that challenge hepatic and renal reserves.

Consequently, these outcomes underscore the urgent necessity of shifting from reactive clinical management toward proactive, individualized risk mitigation. Structured, multidisciplinary clinical pharmacy reviews must be systematically integrated directly into oncology admission protocols. These specialized interventions should be double-pronged: Targeted Nephroprotective Surveillance: Pharmacists must actively screen for, flag, and modulate highly nephrotoxic drug combinations—particularly where platinum-based therapies or intense supportive regimens intersect—to protect renal clearance and stabilize baseline creatinine levels. Pre-Therapeutic Companion Genotyping: Given the high utilization of fluoropyrimidines and irinotecan in gastrointestinal and colorectal malignancies within this tertiary care setting, integrating companion pharmacogenetic screening is highly warranted. Preemptively establishing a patient’s *DPYD* and *UGT1A1* polymorphic status allows for precise, genome-guided dose titrations before initial drug exposure. By combining automated DDI screening with genetic profiling and comprehensive over-the-counter herbal supplement reconciliation, clinical teams can safely deliver core antineoplastic regimens, significantly reduce preventable iatrogenic morbidity, and ultimately improve discharge outcomes and lower the in-hospital mortality proportion in hospitalized cancer patients.

## Data Availability

The original contributions presented in the study are included in the article/Supplementary Material, further inquiries can be directed to the corresponding author.
