# Supplementary material for: Clinical, laboratory, and medication-related factors associated with in-hospital mortality among oncology patients at a tertiary hospital in Saudi Arabia
Source: Front Pharmacol. 2026 Aug 28;17:1825631. doi: 10.3389/fphar.2026.1825631 (PMC13561838; doi:10.3389/fphar.2026.1825631)
Supplement: Supplementary file 1 [file Table1.docx]

**Supplementary File S1: *Data Completeness and Missingness Across Cohort Variables (***$\boldsymbol{N=401}$***)***

| Variable Category | Parameter | Total Recorded (n) | Missing Values  (n,[%]) |
| --- | --- | --- | --- |
| Demographics | Sex / Age / Nationality | 401 | 0 (0.0%) |
| Vital Signs | Oxygen Saturation (SpO2) | 311 | 90 (22.4%) |
|  | Systolic / Diastolic BP | 385 | 16 (4.0%) |
| Laboratory Parameters | Serum Albumin | 401 | 0 (0.0%) |
|  | Serum Creatinine | 401 | 0 (0.0%) |
|  | INR | 352 | 49 (12.2%) |

**S1. Utilization pattern as combination protocol in the oncological treatment:**

Crucially, clinical audit of the chemotherapy sub-cohort revealed high compliance with evidence-based guidelines for gastrointestinal and colorectal malignancies, characterized by the robust representation of 5-fluorouracil (5-FU), oxaliplatin, and irinotecan ($n=174$ total prescriptions). Rather than isolated monotherapies, these cornerstone agents were predominantly utilized within validated combination protocols. Standard frontline regimens included FOLFOX variants (44 prescriptions), FOLFIRI combinations (36 prescriptions), and XELOX/CapOx protocols (38 prescriptions). Intensive multi-agent therapies for advanced or metastatic disease, such as FOLFOXIRI, FOLFIRINOX, and combinations paired with monoclonal antibodies (e.g., Bevacizumab, Cetuximab, or Panitumumab), accounted for an additional 8 prescriptions. Standalone or sequential monitoring orders for oxaliplatin and irinotecan constituted the remaining 48 entries. This distribution confirms that standard-of-care recommendations for these high-priority therapeutic classes were actively tracked and embedded within the studied institutional clinical paths. Table S1

**Table S1.** Distribution and clinical utilization of guideline-recommended chemotherapy regimens and protocols for gastrointestinal and colorectal malignancies ($N=174$ prescriptions).

| Regimen/Drug Profile | Specific Documentation in Dataset (Drug_name_given) | Number  of Prescriptions (n) |  |
| --- | --- | --- | --- |
| FOLFOX Regimens | FOLFOX6 (n=29), FOLFOX (n=11), FOLFOX4 (n=2), FOLFOX + Bevacizumab (n=1), Oxaliplatin + 5-FU + Cetuximab (n=1) | 44 |  |
| FOLFIRI Regimens | FOLFIRI (n=31), FOLFIRI + Panitumumab (n=2), FOLFIRI + Bevacizumab (n=2), FOLFIRI + Biologics (n=1) | 36 |  |
| XELOX / CapOx Regimens | XELOX (n=30), CapOx (n=3), Oxaliplatin + Capecitabine (n=3), Capecitabine + Oxaliplatin (n=1), CapOx + Panitumumab (n=1) | 38 |  |
| Advanced Combination Regimens | FOLFIRINOX (n=2), FOLFOXIRI (n=2), FOLFIRI + XELOX (n=1), FOLFOX6 + FOLFIRI (n=1), FOLFOX/XELOX + Nivolumab (n=1), Gemcitabine + Oxaliplatin + Capecitabine (n=1) | 8 |  |
| Standalone/Isolate Orders | Standalone Oxaliplatin (n=28), Standalone Irinotecan (n=17), FU/LV (n=2), Standalone 5-FU (n=1) | 48 |  |
| Total Prescriptions Utilizing These Agents | — | 174 |  |

*The total of 174 prescriptions was calculated by adding the individual entries from your text: 44 FOLFOX + 36 FOLFIRI + 38 XELOX + 8 multi-agent/mAbs + 48 oxaliplatin/irinotecan monitoring orders*
